# Metabolomics Based Identification of SIRT5 and Protein Kinase C Epsilon Regulated Pathways in Brain

**DOI:** 10.3389/fnins.2018.00032

**Published:** 2018-01-30

**Authors:** Kevin B. Koronowski, Nathalie Khoury, Kahlilia C. Morris-Blanco, Holly M. Stradecki-Cohan, Timothy J. Garrett, Miguel A. Perez-Pinzon

**Affiliations:** ^1^Cerebral Vascular Disease Research Laboratories, Miller School of Medicine, University of Miami, Miami, FL, United States; ^2^Neuroscience Program, Miller School of Medicine, University of Miami, Miami, FL, United States; ^3^Department of Neurology, Miller School of Medicine, University of Miami, Miami, FL, United States; ^4^Southeast Center for Integrated Metabolomics, Clinical and Translational Science Institute, University of Florida, Gainesville, FL, United States

**Keywords:** brain metabolism, cerebral ischemia, ischemic preconditioning, protein kinase C epsilon, SIRT5, sirtuins

## Abstract

The role of Sirtuins in brain function is emerging, yet little is known about SIRT5 in this domain. Our previous work demonstrates that protein kinase C epsilon (PKCε)-induced protection from focal ischemia is lost in SIRT5^−/−^ mice. Thus, metabolic regulation by SIRT5 contributes significantly to ischemic tolerance. The aim of this study was to identify the SIRT5-regulated metabolic pathways in the brain and determine which of those pathways are linked to PKCε. Our results show SIRT5 is primarily expressed in neurons and endothelial cells in the brain, with mitochondrial and extra-mitochondrial localization. Pathway and enrichment analysis of non-targeted primary metabolite profiles from Sirt5^−/−^ cortex revealed alterations in several pathways including purine metabolism (urea, adenosine, adenine, xanthine), nitrogen metabolism (glutamic acid, glycine), and malate-aspartate shuttle (malic acid, glutamic acid). Additionally, perturbations in β-oxidation and carnitine transferase (pentadecanoic acid, heptadecanoic acid) and glutamate transport and glutamine synthetase (urea, xylitol, adenine, adenosine, glycine, glutamic acid) were predicted. Metabolite changes in SIRT5^−/−^ coincided with alterations in expression of amino acid (SLC7A5, SLC7A7) and glutamate (EAAT2) transport proteins as well as key enzymes in purine (PRPS1, PPAT), fatty acid (ACADS, HADHB), glutamine-glutamate (GAD1, GLUD1), and malate-aspartate shuttle (MDH1) metabolic pathways. Moreover, PKCε activation induced alternations in purine metabolites (urea, glutamine) that overlapped with putative SIRT5 pathways in WT but not in SIRT5^−/−^ mice. Finally, we found that purine metabolism is a common metabolic pathway regulated by SIRT5, PKCε and ischemic preconditioning. These results implicate Sirt5 in the regulation of pathways central to brain metabolism, with links to ischemic tolerance.

## Introduction

The silent information regulator two homolog proteins (Sirtuins) are an evolutionarily conserved family of NAD^+^-dependent deacylases that regulate metabolic homeostasis in response to low nutrient availability and energetic stress (Houtkooper et al., 2012). There are seven mammalian Sirtuins (SIRT1-7), 3 of which (SIRT3-5) are primarily localized in, but not limited to, the mitochondrion (He et al., 2012). SIRT5 function is distinct from SIRT3, the major mitochondrial deacetylase, and SIRT4, which mediates ADP-ribosylation and has lipoamidase activity, in that it acts specifically on succinyl- (Rardin et al., 2013), malonyl- (Nishida et al., 2015), and glutaryl- (Tan et al., 2014) lysine post-translational modifications. Metabolic pathways enriched with these modifications and with protein targets of SIRT5 include those central to liver function such as the urea cycle, beta-oxidation and ketone body synthesis, among others (Hirschey and Zhao, 2015). Still, little is known about the functions of SIRT5 in other highly metabolically active tissues such as brain, where cellular metabolism is in large metabolically tailored toward oxidative energy metabolism and the assurance of neurotransmission. Identifying the tissue-specific functions of SIRT5 and other sirtuins is essential to understand their global functionality under physiological and pathological conditions.

Our recent work implicates SIRT5 in preconditioning-induced neuroprotection from cerebral ischemia. Ischemic preconditioning (IPC), an intervention that promotes mitochondrial integrity (Thompson et al., 2015), refers to the ability of a non-injurious bout of ischemia to protect against a subsequent, damaging ischemic injury (Dirnagl et al., 2009). IPC induces innate protective pathways that culminate in an ischemia-tolerant phenotype; we have previously demonstrated that both Sirtuins (Raval et al., 2006) and protein kinase C epsilon (PKCε) (Morris-Blanco et al., 2014), a calcium-independent serine/threonine kinase, are required for IPC-induced ischemic neuroprotection. Both PKCε activation and IPC increase desuccinylation activity in mitochondria, suggesting activation of SIRT5 (Morris-Blanco et al., 2016). Strikingly, PKCε activation is sufficient to protect WT mice against ischemic stroke *in vivo*, yet this protection is lost in SIRT5^−/−^ mice (Morris-Blanco et al., 2016). Therefore, metabolic regulation by SIRT5 contributes significantly to ischemic tolerance in the brain. Despite this finding, the metabolic pathways regulated by SIRT5 in the brain are not known and are likely distinct from those identified in liver (Rardin et al., 2013; Tan et al., 2014; Nishida et al., 2015) and heart (Sadhukhan et al., 2016).

The goal of the present study was two-fold. First, we sought to identify the SIRT5-regulated metabolic pathways in brain and second, to determine which of these pathways are induced downstream of PKCε. To this end, we characterized SIRT5 protein localization in the brain and conducted non-targeted, primary metabolomics analysis of WT and SIRT5^−/−^ cortex, with and without PKCε activation. Furthermore, we investigated transcriptional alterations of key metabolic transporters and enzymes in putative SIRT5-regulated pathways. Finally, we conducted analysis integrating metabolomic and transcriptomic data to identify common metabolic pathways induced across ischemic and pharmacological preconditioning strategies.

## Materials and methods

### Animals and pharmacological treatments

All animal usage and experimentation was approved by the Institutional Animal Care and Use Committee at the University of Miami and was in accordance with the Guide for the Care and Use of Laboratory Animals published by the National Institutes of Health. Reporting is in compliance with ARRIVE (Animal Research: Reporting *in vivo* Experiments) guidelines. Eight- to twelve-week-old adult WT 129S1/SvlmJ (stock no: 002448) and SIRT5^−/−^ (stock no: 012757) male mice (total *n* = 32) were obtained from The Jackson Laboratory (Bar Harbor, ME, USA). Mice were housed at 25°C in a supervised facility of the division of veterinary resources 2–5/cage (only after injury were they housed singly for 24 h) on a 12 h light/dark cycle with ad libitum access to standard chow and water. Mice were injected i.p. with 0.75 mg/kg ΨεRACK (PKCε activator) or Tat (control peptide). Forty-eight hours later, tissues were harvested for metabolomics analysis as described below.

### Immunofluorescence

Mice were anesthetized with 1% isoflurane and perfused with cold saline followed by 4% paraformaldehyde in PBS (pH 7.4). The brain was removed and placed in 4% paraformaldehyde overnight at 4C. Twenty-four hours later, the brain was placed in 20% sucrose solution for up to 2 days. Sagittal 30 μM cryo-sections were cut with a CM-1850 cryostat (Leica, Wetzlar, Germany). Free floating sections were permeabilized and blocked simultaneously for 1 h at RT using 20% Goat serum and 0.2% triton X in antibody buffer (NaCl 150 mM, Tris Base 50 mM, BSA 1%, L-Lysine 100 mM, sodium azide 0.04%, pH 7.4). Sections were then washed three times with PBS and incubated with primary antibodies (see Table S1) overnight at 4°C. Following another 3 washes, sections were incubated with secondary antibodies (Alexa Fluor 488, 568 or 635, 1:500, Life Technologies, Carlsbad, CA, USA) for 2 h at RT. Sections were imaged on an Olympus FV1000 confocal microscope (Center Valley, PA, USA) and rendered onFV10-ASW 2.0 Viewer. Brightness was adjusted equally to all channels across images in PowerPoint software (Microsoft, Redmond, WA).

### Subcellular fractionation

**Figure 1:**
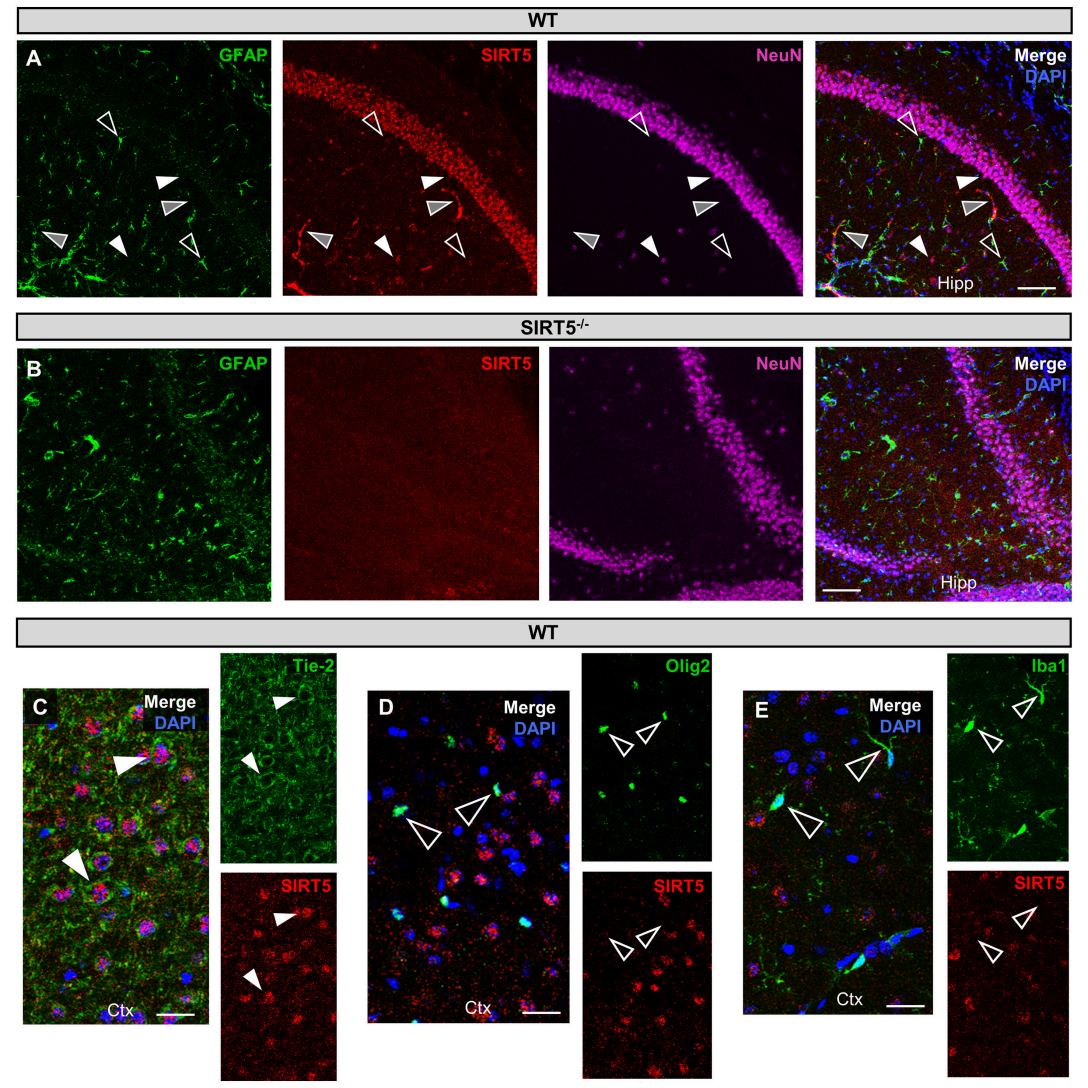
SIRT5 cell types in brain. **(A–E)** Confocal immunofluorescence images from cryo-sections of WT or SIRT5^−/−^ brains. Staining was confirmed across multiple sections and in separate mice. Solid white triangles point to examples of co-localization with SIRT5. Open triangles show examples of no co-localization. Solid gray triangles indicate co-localization with an un-stained cell type. Far-red fluorescence was pseudo-colored magenta. **(A)** WT sections; GFAP, glial marker; NeuN, neuronal marker; in hippocampus (Hipp), Scale bar = 100 μM. **(B)** Same as A but in SIRT5^−/−^ sections, where no SIRT5 positive cells are found. **(C–E)** WT sections; Tie-2, endothelial marker; Olig2, oligodendrocyte marker; Iba1, microglial marker; in cortex (Ctx), Scale bar = 50 μM.

Mice were anesthetized with 1% isoflurane and decapitated. Brains were dissected and cortical tissue was fractionated into subcellular compartments as described previously (Dimauro et al., 2012), with slight modifications. Briefly, tissue was homogenized into a single cell suspension in STM buffer (250 mM sucrose, 50 mM Tris-HCl, 5 mM MgCl_2_, pH 7.4, supplemented with Protease Inhibitor Cocktail (Sigma) and phosSTOP (Roche). Times and speeds of centrifugation steps are depicted in Figure 1 of the cited reference. Sonication steps were omitted. Instead, once purified fractions were obtained they were lysed in RIPA buffer for 30 min on ice and then centrifuged at 12,000 rpm for 20 min. The supernatant was then collected for protein concentration measurements as described below in the western blot analysis section.

### Western blot analysis

Protein concentration was determined by the BioRad DC™ Protein Assay. Forty micrograms of protein from each fraction was loaded into a 4–20% SDS polyacrylamide gel (BioRad, Hercules, CA, USA). Proteins were then transferred onto a nitrocellulose membrane (Biorad) and blocked for 2 h at RT (5% milk in TBS-T, 0.1% Tween-20 in PBS). Incubation in primary antibody was achieved overnight at 4°C. Please see Table S1 for specific primary antibody information. Following addition of HRP-conjugated secondary antibodies (1:5,000, GE healthcare, Little Chalfont, UK) for 1 h, blots were developed with ECL reagents (ThermoFisher Scientific, Waltham, MA, USA) and visualized with X-ray film (Denville Scientific, Holliston, MA). Blots were then digitalized and analyzed in Image J software (NIH) by densitometry.

### Non-targeted primary metabolomics

Mice were anesthetized with 1% isoflurane, perfused with cold saline and then decapitated. Subsequently, brains were dissected and cortices were snap frozen in liquid nitrogen. After this step, sample preparation, data acquisition, and data processing was performed by the West Coast Metabolomics Center (UC-Davis) as previously described (Fiehn et al., 2008). Briefly, this non-targeted primary metabolism platform was achieved by gas chromatography—time of flight—mass spectrometry (GC-TOF-MS). Mass spectrometer (Leco Pegasus IV, St. Joseph, MI) settings: unit-mass resolution at 17 spectra s^−1^ from 80 to 500 Da at −70 eV ionization energy and 1800 V detector voltage with 230°C transfer line and 250°C ion source. Data processing: ChromaTOF vs. 2.32 without smoothing, a 3 s peak width, baseline subtraction above noise level, automatic mass spectral deconvolution and peak detection at 5:1 (signal/noise).

### Metabolomics statistical, pathway, and enrichment analyses

Peak intensity values normalized to tissue weight were used for statistical analysis in MetaboAnalyst 3.0 (Xia and Wishart, 2016). For normalization, values were log transformed and auto scaled. No filtering was applied given the relatively small number of features identified. Compound names were used for input into pathway and enrichment analysis tools. For Pathway Analysis, which integrates enrichment and pathway topology, over representation was achieved by Fisher's Exact Test and pathway topology by out-degree centrality. Two analyses were run in Enrichment Analysis, which performs metabolite set enrichment analysis (MSEA). Metabolites were compared with (1) pathway-associated metabolite sets, based on normal metabolic pathways and (2) predicted metabolite sets, containing metabolites predicted to be changed in the case of dysfunctional enzymes using genome-scale network model of human metabolism. Integrated Pathway Analysis was carried out using hypergeometric test for enrichment, degree centrality for topology and the gene-metabolite database option. Measured metabolite names were used as input for MetaMapR (Grapov et al., 2015), to build a network that displays structural similarity of metabolites based on their PubChem substructure fingerprints. The network was then exported and visualized using Cytoscape (Kohl et al., 2011). All structurally annotated metabolites measured were used to generate structural connections in MetaMapR; however, not all metabolites had structural connections and thus not all metabolites are represented in the network. The full list of metabolites and their metrics can be found in Table S1.

### Real-time quantitative PCR

Real-time qPCR was conducted as described previously (Morris-Blanco et al., 2016). In short, TRIzol (Life Technologies) homogenized hippocampi, from the same mice as metabolomics samples, were used for RNA extraction by RNeasy Mini Kit (Qiagen, Hilden, Germany). One microgram of RNA was used as the template for cDNA synthesis by qScript cDNA SuperMix (Quanta Biosciences, Beverly MA, USA). Real-time qPCR was carried out with Power SYBR Green Master Mix (ThermoFisher Scientific) in triplicate using the LightCycler® 480 II (Roche Applied Science, Penzberg, Germany). Results were analyzed by the ΔΔCT method and normalized as fold change of control. For primer sequences, see Table S1.

### Statistical analysis

Mice were assigned treatment groups by block randomization in Excel (Microsoft). All data acquisition and analyses were done in a blinded manner. Statistical analysis was performed in Prism6 software (GraphPad, San Diego, CA). Data are presented as mean ± SEM unless otherwise stated. Two-sample, unpaired student's *t*-test was used as indicated in the text and figure legends. Pathway and enrichment significance cutoff values are listed in the text. Sample size was determined by power analysis in order to detect an effect size of 0.8 using G^*^power 3.1 software.

## Results

### SIRT5 protein expression and subcellular localization in brain

**Figure 2:**
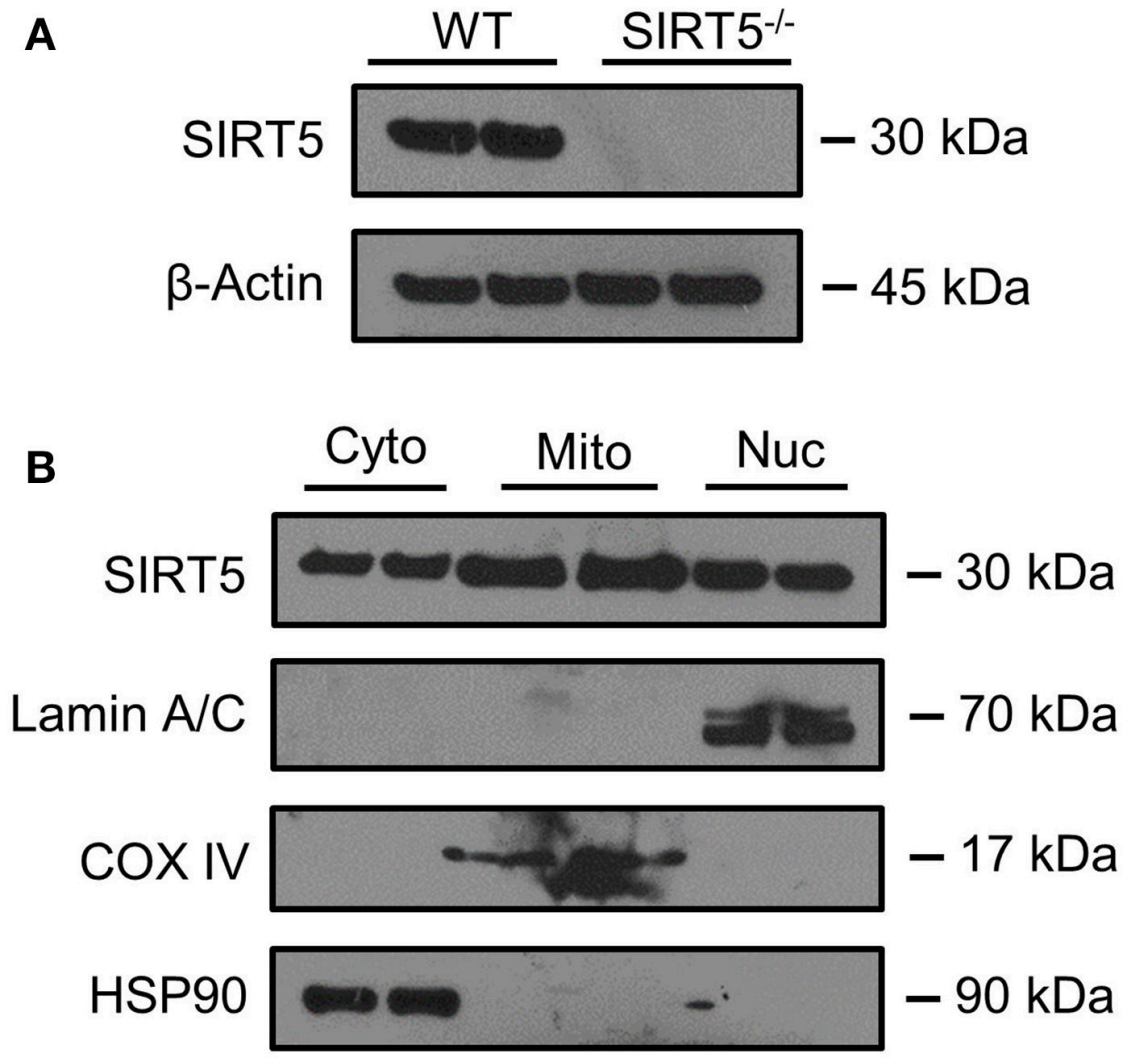
SIRT5 Subcellular localization in brain. **(A,B)** Western blot analysis of SIRT5 protein from brain. **(A)** No SIRT5 protein was observed in SIRT5^−/−^ hippocampus, demonstrating specificity of the antibody. **(B)** Cytosolic (Cyto), mitochondrial (Mito), and nuclear (Nuc) fractions from WT cortical brain tissue are positive for SIRT5 protein (2 biological replicates are depicted, *n* = 4). Compartment-specific markers are overexposed to highlight fraction purity. Images are cropped for clarity; Lamin A/C, nuclear; COX IV, mitochondrial; HSP90, cytosolic.

SIRT5 expression is abundant in the developed brain (Zhang et al., 2014), yet its localization is unknown. Immunostaining for SIRT5 in adult WT mice revealed a predominantly neuronal-like expression pattern in hippocampus and cortex, evidenced by co-localization with the neuronal marker NeuN but not the glial marker GFAP (Figure 1A). No SIRT5 staining was observed in SIRT5^−/−^ mice, demonstrating specificity of the SIRT5 antibody (Figure 1B). In WT, SIRT5 staining was also present within micro-vessel structures and displayed endothelial-like morphology (Figure 1A). Indeed, significant co-localizaiton of SIRT5 and the endothelial marker Tie-2 was observed (Figure 1C). No SIRT5 co-localization with the oligodendrocyte marker Olig2 (Figure 1D) or the microglial marker Iba1 (Figure 1E) was detected, indicating lack of SIRT5 protein expression within these cell types. Furthermore, by comparing SIRT5 staining with that of NeuN and the nuclear marker DAPI, it appeared to be associated with the soma of cells. To determine more specifically the cellular distribution of SIRT5, we fractionated cortical tissue from WT mice into cytosolic, mitochondrial, and nuclear compartments and performed western blot analysis. No SIRT5 protein was detected in whole-cell lysates from SIRT5^−/−^ brain, demonstrating specificity of this antibody (Figure 2A). In WT, subcellular fractions displayed high purity, evidenced by the localization of compartment-specific markers (Figure 2B). Intriguingly, all three fractions were positive for SIRT5 protein, implicating SIRT5 function in the cytosol and nucleus, in addition to mitochondrion (Figure 2B). Together, these data suggest SIRT5 may regulate mitochondrial and extra-mitochondrial metabolic processes in brain endothelial and neuronal cells.

### Altered metabolic pathways in SIRT5^−/−^ cortex

**Figure 3:**
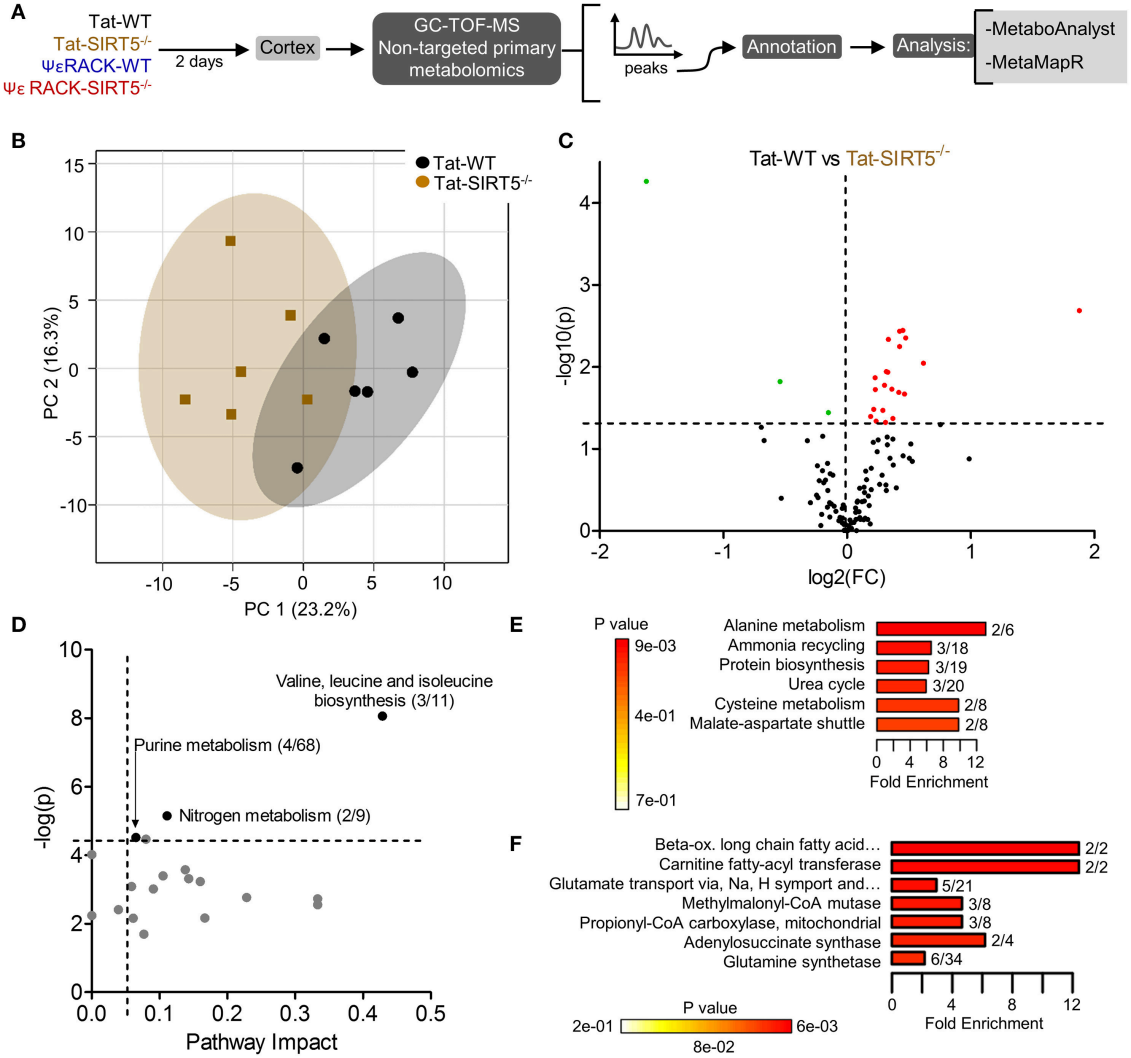
Altered metabolic pathways in SIRT5^−/−^ cortex. **(A)** Schematic of experimental paradigm. Tat, control peptide; ΨεRACK, PKCε-specific activator **(B–F)** comparison of WT and SIRT5^−/−^ cortical metabolite profiles in the control condition (*n* = 6). **(B)** Scores plot from principal component analysis (PCA) showing clustering of groups based on quantitative variances of annotated metabolites. 95% confidence intervals are displayed for each group. **(C)** Volcano plot of –log(*p*-value) and log2(fold-change) values for all annotated metabolites. Dashed lines display significance cutoffs (see text). Green, decrease; Red, increase. **(D–F)** Pathway and enrichment analysis for significantly altered metabolites in SIRT5^−/−^ cortex. **(D)** Higher pathway impact value indicates greater centrality or importance in that particular pathway. Dashed lines display significance cutoffs (see text). **(E)** Enrichment analysis for metabolite sets based on normal metabolic function. Fold enrichment values indicate whether metabolites appear more than expected by random chance (see text for significance cutoffs). **(F)** Same as in E but for metabolite sets predicted to be altered in the case of dysfunctional enzymes.

The metabolic pathways regulated by SIRT5 in the brain are largely unknown. Given that SIRT5 is required for PKCε-induced ischemic tolerance (Morris-Blanco et al., 2016), these pathways are likely contributors to neuroprotection. Here, we performed non-targeted primary metabolomics analysis of adult WT and SIRT5^−/−^ mouse brain with and without PKCε activation. Mice were treated with the PKCε-specific activator ΨεRACK (0.75 mg/kg) or Tat control peptide (*n* = 6). Forty-eight hours later, at the time in which ischemic tolerance is robust (Morris-Blanco et al., 2016), cortices were harvested for metabolite analysis by GC-TOF-MS (Figure 3A). In total, 254 peaks were captured, 112 of which are structurally annotated metabolites. To first identify basal metabolic differences and putative SIRT5-regulated pathways, we compared Tat treated WT and SIRT5^−/−^ groups. Principal component analysis (PCA) clustering showed separation between the groups along principal component 1 (PC1, 23.2% of total variance), indicating an overall difference between metabolite profiles (Figure 3B). Furthermore, student's *t*-test revealed 24 significantly altered metabolites in SIRT5^−/−^ cortex compared to WT [*p* < 0.05, false discovery rate (FDR) < 0.23, Figure 3C and Table 1; Table S2].

**Table 1:** Significantly altered, structurally annotated metabolites in SIRT5^−/−^ cortex^*^.

**Metabolite Name**	***p*-value**	**FDR**	**Fold change**
Glycerol-3-galactoside	0.0001	0.0061	0.3246
Adenosine	0.0021	0.0860	3.6774
Xylitol	0.0036	0.0860	1.3679
Pentadecanoic acid	0.0037	0.0860	1.3431
Urea	0.0044	0.0860	1.3890
Lyxitol	0.0046	0.0860	1.2603
Oxalic acid	0.0056	0.0902	1.3425
Hexitol	0.0090	0.1259	1.5355
Butyrolactam NIST	0.0114	0.1299	1.2459
Adenine	0.0116	0.1299	1.2568
Valine	0.0135	0.1376	1.1701
Cholestan-3-ol	0.0151	0.1405	0.6870
Malic acid	0.0167	0.1409	1.2342
Glycine	0.0186	0.1409	1.2861
Stearic acid	0.0189	0.1409	1.1716
Myristic acid	0.0204	0.1410	1.3367
Xanthine	0.0214	0.1410	1.3806
Palmitic acid	0.0329	0.1990	1.1605
Erythritol	0.0338	0.1990	1.2217
Glutamic acid	0.0360	0.2019	0.9009
Heptadecanoic acid	0.0401	0.2138	1.1416
Threonic acid	0.0425	0.2165	1.2934
Isoleucine	0.0458	0.2213	1.1769
Diacetone alcohol NIST	0.0474	0.2213	1.2402

* *Data from non-targeted primary metabolomics analysis of Tat-WT and Tat-SIRT5^−/−^ cortex. Only structurally annotated metabolites are presented. Fold change is with respect to Tat-WT*.

**Figure 4:**
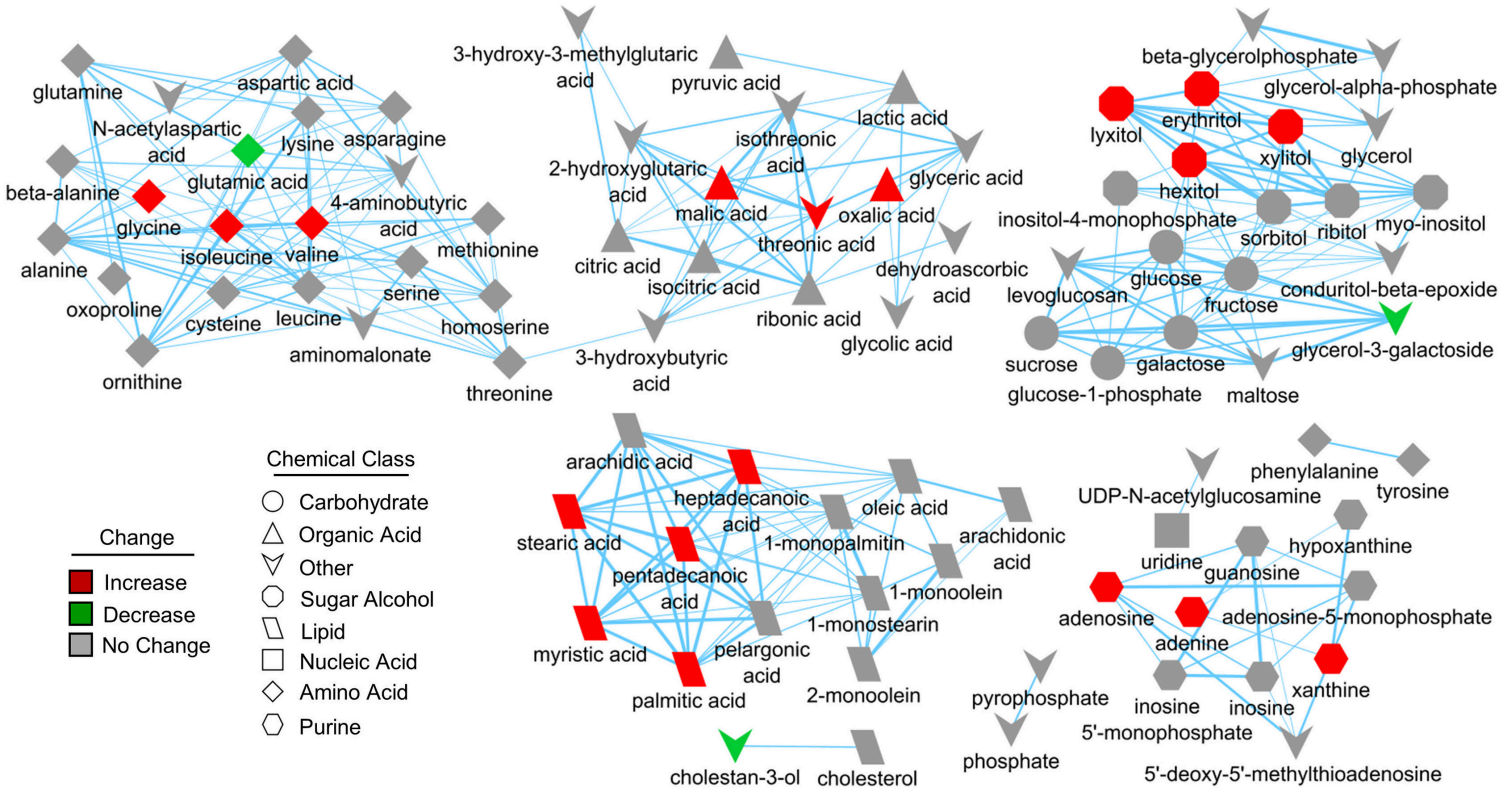
Structural similarity network displaying metabolic perturbations in SIRT5^−/−^ cortex. Metabolites are linked based on structural similarity of PubChem substructure fingerprints. Shape denotes chemical class from KEGG Compound. Relative abundance is represented by color, with respect to Tat-WT. There are several clusters of structurally similar metabolites significantly more abundant in SIRT5^−/−^ cortex.

Further pathway and enrichment analyses were carried out in MetaboAnalyst 3.0 (Xia and Wishart, 2016) to place altered metabolites into their biochemical context. Pathway analysis referencing the KEGG pathway database identified 3 significantly altered pathways, valine, leucine, and isoleucine metabolism (valine, isoleucine), nitrogen metabolism (glutamic acid, glycine), and purine metabolism (xanthine, adenosine, adenine, urea) (≥2 hits, impact > 0.05, *p* ≤ 0.01, Figure 3D). Enrichment analysis referencing libraries of metabolite sets based on normal metabolism found an additional significantly altered pathway with other contributing metabolites [malate-aspartate shuttle (MAS): malic acid, glutamic acid] (≥2 hits, fold enrichment ≥2.0, *p* ≤ 0.01, Figure 3E). Furthermore, analysis based on metabolite sets linked to dysfunctional enzymes suggested possible perturbations in β-oxidation of long chain fatty acids (pentadecanoic acid, heptadecanoic acid), carnitine fatty-acyl transferase (pentadecanoic acid, heptadecanoic acid), glutamate transport via Na^+^/H^+^ symport (urea, adenine, glycine, glutamic acid, adenosine), and glutamine synthetase (xylitol, urea, adenine, glycine, glutamic acid, adenosine) (≥2 hits, fold enrichment ≥2.0, *p* ≤ 0.05, Figure 3F). A structural similarity network was generated in MetaMapR (Grapov et al., 2015) to comprehensively visualize these alterations; from this illustration, it is evident there are several chemical classes of metabolites more abundant in SIRT5^−/−^ including fatty acids, purines, sugar alcohols, organic acids, and amino acids (Figure 4). Together, these results implicate SIRT5 in the regulation of several metabolic pathways in brain.

### Transcriptional changes of metabolic genes in SIRT5^−/−^ cortex

**Figure 5:**
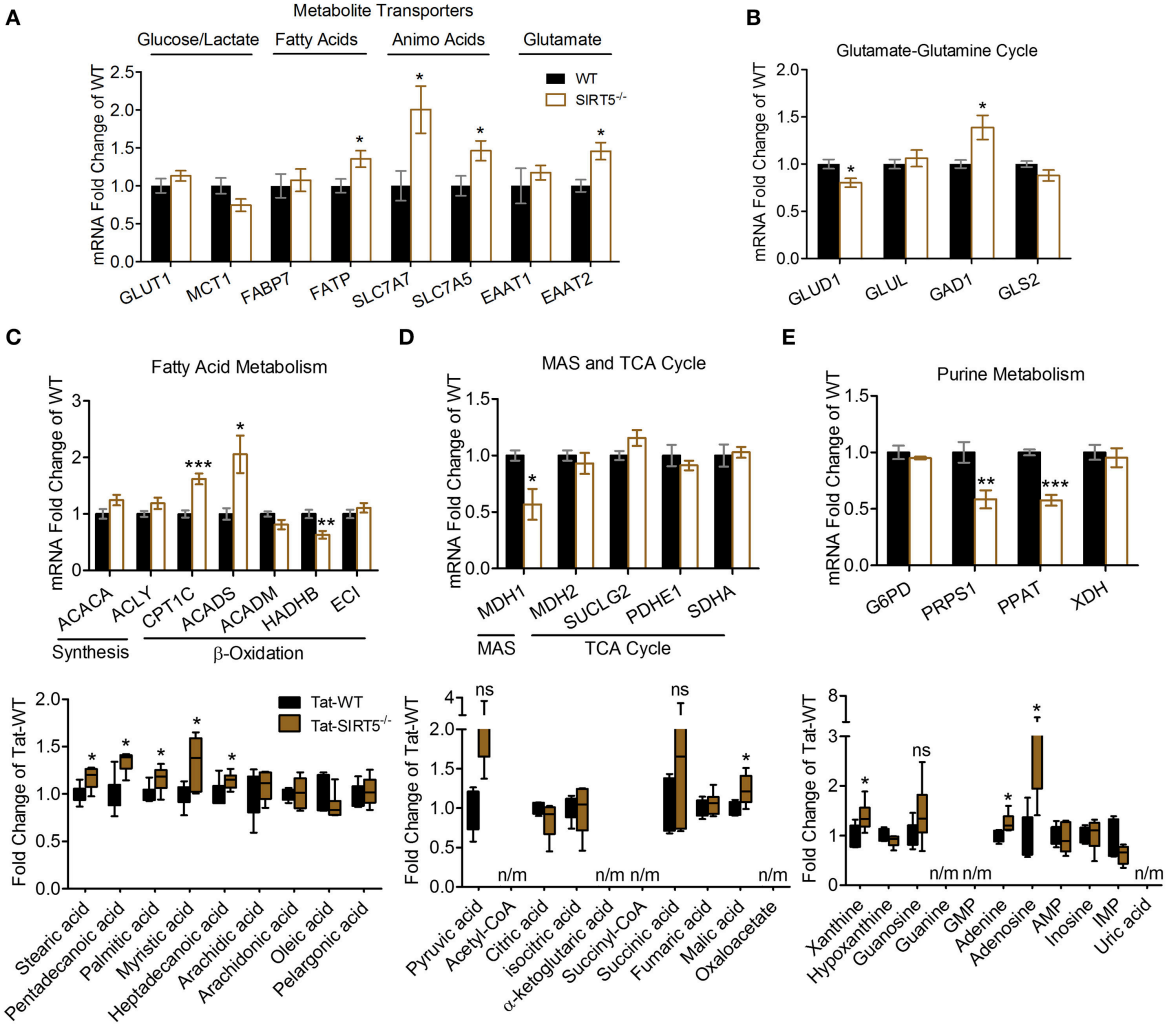
Transcriptional changes of metabolic pathways in SIRT5^−/−^ brain. **(A–E)** Real-time quantitative PCR analysis of gene expression in WT and SIRT5^−/−^ hippocampus of mice used for metabolomics analysis. Expression is presented as mRNA levels fold change of WT. Pathways are indicated at the top (and/or bottom) of each panel. ^*^p < 0.05; ^**^p < 0.01; ^***^p < 0.001; student's *t*-test; *n* = 5–6. ^*^Metabolite significance from initial analysis; ns, not significant; n/m, not measured. **(A)** Transporters for different metabolite classes. **(B)** Rate-limiting and key enzymes for glutamate-glutamine cycle. **(C–E)** Rate-limiting and key enzymes (top) shown with metabolite changes in particular metabolic pathways (bottom, error bars show min and max values of box and whisker blot). **(C)** Fatty acid metabolism. **(D)** Malate aspartate shuttle (MAS), and tricarboxylic acid (TCA) cycle. **(E)** Purine metabolism.

We sought to validate some of the most robustly altered metabolic pathways from our initial analysis. Changes in metabolite abundance observed in SIRT5^−/−^ cortex may reflect increased or decreased uptake into the brain. Thus, we measured expression of key transporter genes linked to glucose, fatty acids and amino acids in hippocampal tissue from the same mice as in the metabolomics analysis. Although not an ideal comparison, these regions appear comparable as they display a similar pattern and intensity of SIRT5 expression and are both protected from ischemia by PKCε *in vivo* (Della-Morte et al., 2011; Neumann et al., 2015). While no differences were observed for glucose transporter 1 (GLUT1, *p* > 0.05) or the pyruvate/lactate carrier monocarboxylate transporter 1 (MCT1, *p* > 0.05), increases in fatty acid transport protein (FATP, +35.43% ± 9.95, *p* < 0.05), Y+L amino acid transporter 1 (SLC7A7, +100.45% ± 32.10, *p* < 0.05), large neutral amino acid transporter (SLC7A5, +46.19% ± 14.58, *p* < 0.05) and excitatory amino acid transporter 2 (EAAT2, +45.68% ± 12.52, *p* < 0.05) were detected (*n* = 5–6, *t*-test, Figure 5A). In the absence of SIRT5, metabolite abundance will also reflect alternations in enzymatic activity, either from hyper-succinyl-, malonyl-, and glutaryl-ation or genetic compensation in enzyme expression. Hence, we measured expression of key enzymatic genes in putative SIRT5-regulated pathways. Enzymes involved in the glutamate-glutamine cycle, glutamate dehydrogenase 1 (GLUD1, −19.80% ± 4.82, *p* < 0.05) and glutamate decarboxylate 1 (GAD1, +38.73% ± 14.32, *p* < 0.05), were significantly altered in SIRT5^−/−^ (*n* = 5–6, *t*-test, Figure 5B). Whereas levels of regulatory fatty acid synthesis enzymes acetyl-CoA carboxylase 1 (ACACA) and ATP citrate lyase (ACLY) were similar (*p* > 0.05), several alterations were found for key fatty acid β-oxidation enzymes, including carnitine palmitoyltransferase 1 (CPT1C, +61.93 ± 10.44, *p* < 0.001), acyl-CoA dehydrogenase short chain (ACADS, +105.58 ± 33.40, *p* < 0.05) and 3-ketoacyl-CoA thiolase (HADHB, −36.79% ± 7.47, *p* < 0.01) (*n* = 5–6, *t*-test, Figure 5C). Of the TCA cycle enzymes that are possibly linked to the observed metabolite changes, malate dehydrogenase 1 (MDH1, −43.23% ± 15.11, *p* < 0.05), the malate aspartate shuttle (MAS) component, but not MDH2 (*p* > 0.05), the TCA cycle component, was decreased (*n* = 5–6, *t*-test, Figure 5D). Furthermore, two regulatory purine biosynthesis enzymes, phosphoribosyl pyrophosphate synthetase (PRPS1, −41.54% ± 3.71, *p* < 0.05) and phosphoribosyl pyrophosphate amidotransferase (PPAT, −42.36% ± 4.14, *p* < 0.05), were also decreased (*n* = 5–6, *t*-test, Figure 5E). Taken together, these data suggest a functional consequence for these central metabolic pathways in the absence of SIRT5.

### Metabolic pathways induced by PKCε activation

**Figure 6:**
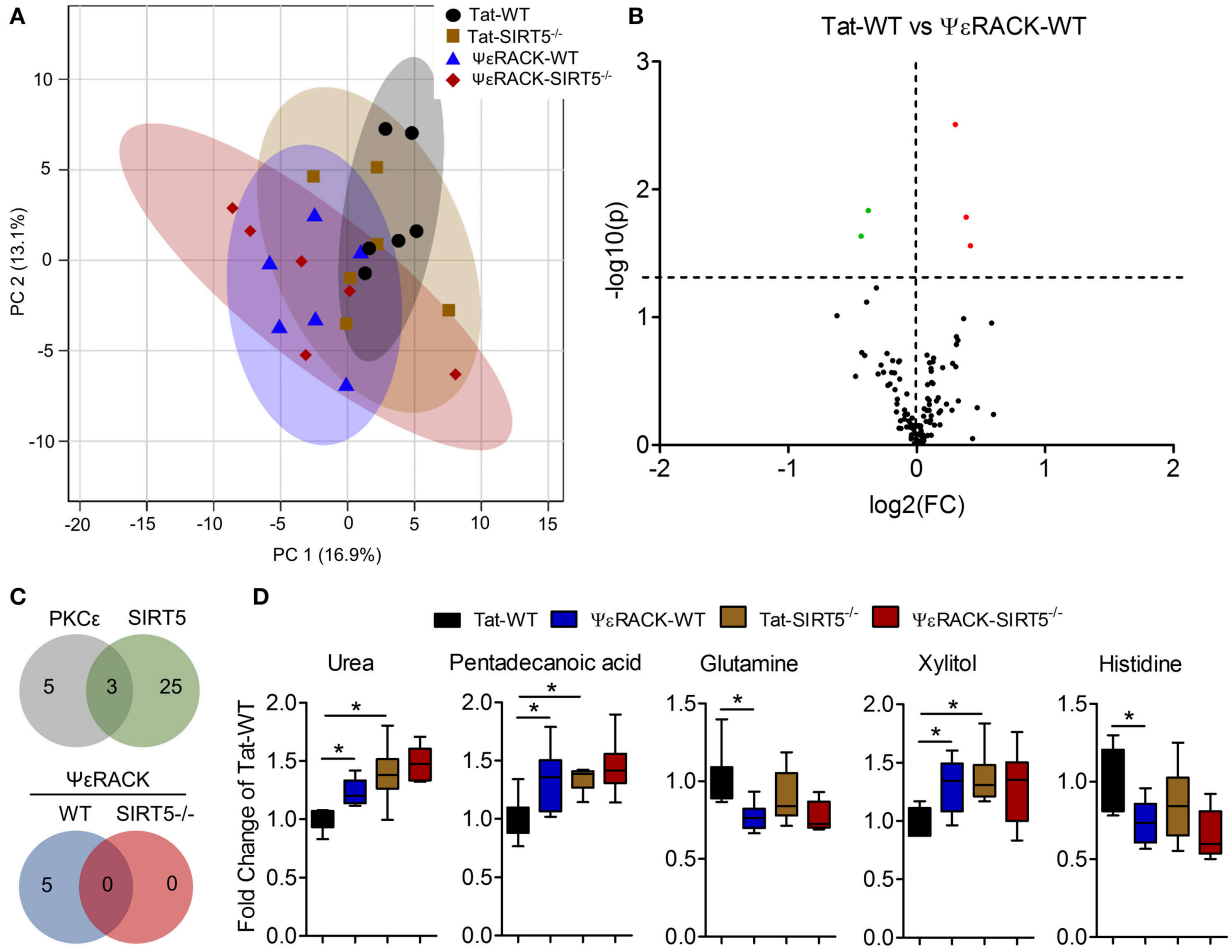
Metabolic alterations induced by PKCε activation. Experimental paradigm is as previously depicted in Figure 3A. **(A)** Principal component analysis (PCA) of PKCε activation in WT or SIRT5^−/−^ mice; Tat, control peptide; ΨεRACK, PKCε-specific activator. The scores plot shows clustering of groups based on quantitative variances of annotated metabolites. 95% confidence intervals are displayed for each group. **(B)** Volcano plot of –log(*p*-value) and log2(fold-change) values for all annotated metabolites comparing PKCε activation in WT. Dashed lines display significance cutoffs (see text). Green, decrease; Red, increase. **(C)** Venn diagrams illustrating the overlap of altered metabolites between conditions. Top, PKCε activation altered the abundance of 5 metabolites, 3 of which were altered by SIRT5 knockout. Bottom, PKCε activation did not alter the abundance of any metabolites in SIRT5^−/−^. **(D)** Relative abundances are shown with respect to Tat-WT for metabolites significantly altered by PKCε activation (error bars show min and max values of box and whisker plot). ^*^Significance from initial student's *t*-tests.

Next, we wanted to determine which metabolic pathways are induced by PKCε, which have implications in ischemic tolerance, and determine which of those pathways are dependent upon SIRT5. To this end, we compared the effects of PKCε activation on the metabolic profiles of both WT and SIRT5^−/−^ cortex. PCA clustering of all four groups showed a small amount of separation along PC1 (16.9% of total variance), indicating a weak overall difference between all metabolite profiles (Figure 6A). When comparing Tat to ΨεRACK-treated WT's, student's *t*-test revealed 5 metabolites that were significantly altered (*p* < 0.05, FDR < 0.23, *n* = 6, Figures 6B,D, Table S3). In SIRT5^−/−^, there were no significantly altered metabolites with ΨεRACK treatment, demonstrating that these changes required SIRT5 (Figure 6C—bottom, Table S4). Of the 5 ΨεRACK-induced metabolites, 3 were also altered in SIRT5^−/−^ and 2 were purine metabolites (urea, glutamine), suggesting commonality in PKCε- and SIRT5 regulation of this pathway (Figure 6C, top). These data suggest that PKCε and SIRT5-mediated regulation of purine metabolism may play a role in ischemic tolerance.

### Integrated pathway analysis of preconditioning-induced transcriptomic and metabolomic changes

**Figure 7:**
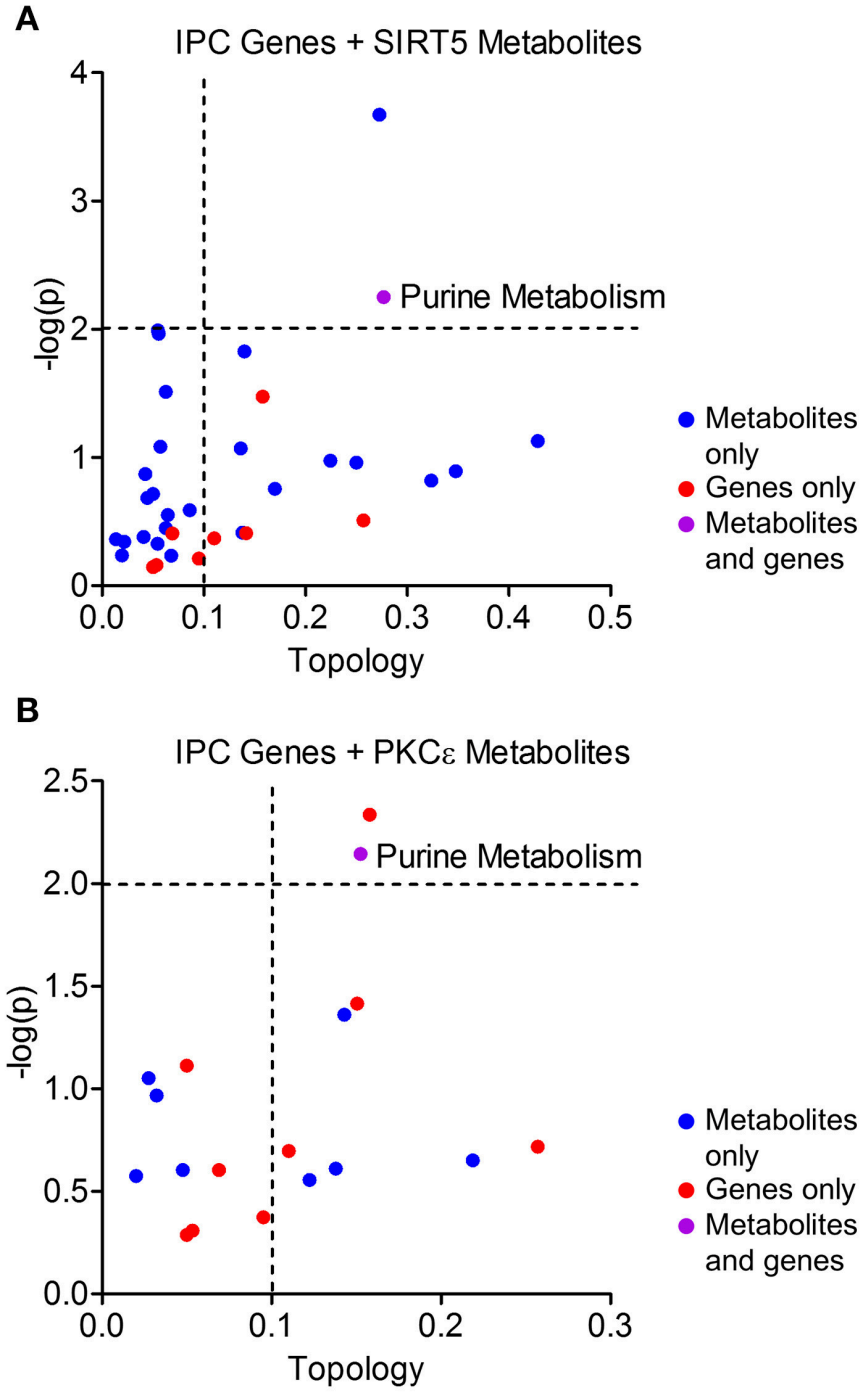
Integrated pathway analysis of ischemic preconditioning-induced genes and PKCε/SIRT5-regulated metabolites. Common metabolic pathways of IPC-regulated genes and SIRT5- or PKCε-regulated metabolites were identified by integrated pathway analysis. Topology values represent the relative importance of a gene or metabolite given its position in a particular pathway (degree centrality). Dashed lines display significance cutoffs (see text). Purple coloring represents pathways enriched with both genes and metabolites. **(A)** IPC genes and metabolites from SIRT5^−/−^ cortex. **(B)** IPC genes and metabolites from PKCε-activation.

Protective pathways of preconditioning-induced ischemic tolerance have been identified by transcriptomics, proteomics and metabolomics-based data sets (Stenzel-Poore et al., 2003; Scornavacca et al., 2012; Laursen et al., 2017). It is postulated that integration of RNA, protein and metabolite changes culminates in an ischemia tolerant cellular phenotype, yet few studies have attempted to identify the common pathways across omics platforms. Thus, we performed integrated metabolic pathway analysis that incorporates both metabolomics and transcriptomics data to identify common metabolic pathways from RNA to metabolite. Given that IPC requires both PKCε and sirtuins, we analyzed significantly altered metabolic genes 24 or 72 h following ischemic preconditioning in mouse cortex (Stenzel-Poore et al., 2003) with significantly altered metabolites from SIRT5^−/−^ or PKCε activation in the present study. IPC altered the expression of 31 metabolic genes in various pathways including arachidonic acid metabolism, tryptophan metabolism, purine metabolism, metabolism of xenobiotics by cytochrome P450 and pyrimidine metabolism (≥2 hits, see cited reference Table 2, Stenzel-Poore et al., 2003). Integrated pathway analysis identified purine metabolism (genes–*POLR2A, AK1, POLA1*; metabolites–xanthine, urea, adenine, adenosine) as the only pathway enriched for both IPC genes and SIRT5 or PKCε metabolites (*p* < 0.01, topology ≥ 0.15, Figures 7A,B). This analysis suggests that regulation of purine metabolism may be a common pathway of ischemic tolerance that is fully transduced from RNA to metabolite.

## Discussion

Recent studies demonstrate the emerging role of succinyl-, malonyl-, and glutaryl-lysine modifications, and their regulation by SIRT5, within metabolically active tissues such as liver and heart (Hirschey and Zhao, 2015). Yet, the significance of these novel modifications and their regulation by SIRT5 is unclear in the brain, an organ with highly specialized and compartmentalized metabolic pathways. Given that SIRT5 is necessary for PKCε-induced ischemic tolerance against stroke (Morris-Blanco et al., 2016), these metabolic pathways are likely mediators of this protection. In the present study, analysis of metabolite profiles from WT and SIRT5^−/−^ cortex reveal both overlapping and distinct functions of SIRT5 in the brain with respect to liver.

Urea cycle (Tan et al., 2014), ketone body synthesis (Rardin et al., 2013) and β-oxidation (Rardin et al., 2013) are SIRT5-regulated pathways essential to hepatocyte function; however, their significance in brain is uncertain. For example, the urea cycle is not thought to exist in brain, as expression of key enzymes, such as CPS1 (a deglutarylation target of SIRT5), is absent (Kurbat and Lelevich, 2009). Instead, the brain disposes of ammonia through the glutamate-glutamine cycle, whereby glutamine synthetase converts glutamate and ammonia to glutamine (Kosenko et al., 2003). This pathway is also central to the synthesis and recycling of the neurotransmitters glutamate and GABA. Intriguingly, glutamate transport and glutamine synthetase function were predicted to be dysfunctional based on the altered metabolite profile of SIRT5^−/−^ cortex. Glutamate was less abundant in SIRT5^−/−^, concomitant with an increase in EAAT2, the primary glutamate reuptake transporter (Lin et al., 2012). Additionally, glutamine was less abundant with PKCε activation in WT but not SIRT5^−/−^, suggesting regulation of this cycle by SIRT5. In line with this notion, SIRT5^−/−^ displayed altered expression of key enzymes, GLUD1 and GAD1, which may signify genetic compensation for misregulation of this pathway. Importantly, localization of SIRT5 in neurons suggests that glutaminase may be a protein target of SIRT5 rather than glutamine synthetase, which is localized in astrocytes (Martinez-Hernandez et al., 1977). Ammonia is toxic in brain and accumulates during ischemia-reperfusion injury (Holownia et al., 1994). SIRT5 enhancement of ammonia disposal, similar to that in liver, could be a mechanism of significant ischemic protection. However, this remains to be tested functionally.

In both liver (Rardin et al., 2013) and heart (Sadhukhan et al., 2016), succinylated proteins are enriched in pathways pertaining to fatty acid metabolism and more specifically β-oxidation. Oxidation of fatty acids provides an additional fuel source to glucose in these tissues yet its contribution to energy metabolism in the brain is debated. A current view is that fatty acids are not a significant fuel source for the brain, given a semi-restricted fatty acid transport or diffusion across the blood brain barrier (BBB), a lessened capacity of the β-oxidation due to decreased activity or expression of certain enzymes and the potential for severe oxidative stress (Schonfeld and Reiser, 2013). However, a recent estimate of total brain energy expenditure suggests that β-oxidation of fatty acids may account for up to 20% of this rate (Ebert et al., 2003). Previous studies show that SIRT5^−/−^ liver, skeletal muscle and heart accumulate different fatty acid species including medium and long-chain acylcarnitines (Rardin et al., 2013) and long-chain fatty acyl-CoAs (Sadhukhan et al., 2016). Our analysis of SIRT5^−/−^ cortex revealed increased abundance of many saturated fatty acids as well as predicted carnitine fatty acyl transferase and enzymes in β-oxidation of long-chain fatty acids to be dysfunctional. One brain fatty acid transporter, FATP, was modestly increased, although the role of fatty acid transport proteins in brain is poorly understood (Jia et al., 2007). Furthermore, alterations in expression of the key β-oxidation enzymes CPT1C and ACADS were observed, suggesting a potential defect in this pathway. It should be noted that CPT1C, the brain-specific isoform of the CPT1 enzyme, has not been shown to have acyltransferase activity akin to CPT1A-B but rather is involved in brain oxidative metabolism (Lee and Wolfgang, 2012), possibly acting as an energy sensor through malonyl-CoA (Wolfgang et al., 2006). There are several known targets of SIRT5 in heart β-oxidation including enoyl-CoA hydratase, which is activated by SIRT5-mediated desuccinylation (Sadhukhan et al., 2016). Interestingly, it is postulated that if a significant degree of β-oxidation does occurs in brain, it takes place in astrocytes due to their proximity to the BBB and possible role in the uptake of carnitine and unbound long-chain fatty acids from blood (Nalecz et al., 2004). Alternatively, neural stem and progenitor cells of the subventricular and subgranular zones display fatty acid oxidative metabolism, a feature that regulates their proliferative state (Stoll et al., 2015; Knobloch et al., 2017). Yet, these cells are localized to region-specific niches and make up a small percentage of total brain cells. In the present study, the expression of SIRT5 in endothelial cells and neurons raises the possibility of β-oxidation within these compartments. Further studies are needed to investigate the contribution of SIRT5 and β-oxidation to energy metabolism in brain.

Several pieces of evidence suggested perturbations in the MAS and TCA cycle in SIRT5^−/−^ cortex. MAS is the main mechanism by which reducing equivalents (NADH) and NAD^+^ are recycled between the mitochondrion and cytosol (McKenna et al., 2006). Proper function of MAS is essential for the efficiency of TCA cycle and oxidative phosphorylation, which rely heavily on NAD^+^/NADH as a redox co-factor. We observed increased abundance of malate and oxalate (formed from oxaloacetate) that was concomitant with a marked reduction in expression of MDH1, the cytosolic MAS isoform (Musrati et al., 1998) but not the mitochondrial TCA cycle isoform MDH2, suggesting a dysregulation of this process. Mitochondria isolated from SIRT5^−/−^ cortex display a reduction in respiration in the presence of complex I, II, and IV-linked substrates, supporting this notion (Morris-Blanco et al., 2016). Furthermore, PKCε activation 2 days prior to stroke in mice increases respiration at all complexes 2 h after injury, an effect that was lost in SIRT5^−/−^ mice (Morris-Blanco et al., 2016). We have also shown that PKCε enhances respiration of synaptic mitochondria (Dave et al., 2008), implicating these changes in synaptic function. Together, these data suggest that SIRT5 regulates mitochondrial bioenergetics, likely in neurons, to promote ischemic tolerance. While it remains to be seen which substrates are responsible for this enhanced energetic function, it is possible that β-oxidation of fatty acids contributes to these effects, linking SIRT5 regulation of these two pathways.

Importantly, these results in cortex are in stark contrast to the liver, where SIRT5 deficient mitochondria display increased respiration in the presence of complex I and II-linked substrates (Park et al., 2013). Here, the inhibitory effect of SIRT5 on respiratory chain activity is mediated by desuccinylation of pyruvate dehydrogenase (PDH) and succinate dehydrogenase (SDH) complex subunits (Park et al., 2013). Part of this discrepancy could be explained by the fact that functionality of liver mitochondria is highly dependent upon the metabolic state of the liver and whole-organism (Panov et al., 1975). Conversely, the brain requires constant and large respiratory activity of mitochondria in order to meet the metabolic demand, which is somewhat disconnected from the organismal metabolic state by the BBB. Additionally, brain mitochondria contain SDH that is already strongly inhibited by oxaloacetate and malate concentration (Broer et al., 2004; Panov et al., 2009). Further comparison of the metabolic phenotypes of liver vs. brain mitochondria is needed to identify the precise tissue-specific regulation that gives rise to differential respiratory activity.

Another key finding of this study is a potential role for SIRT5 in the regulation of purine metabolism. Purine metabolites are precursors of deoxyribonucleotide triphosphates (dNTPs) and ribonucleotide triphosphates (rNTPs), essential molecules for DNA synthesis and repair, chemical energy transport (i.e., ATP, GTP), neurotransmission and other signaling mechanisms in brain (Dale and Frenguelli, 2012). Their *de novo* synthesis stems from glucose-derived serine and ribose-5-phosphate generated by the pentose phosphate pathway (PPP). SIRT5-mediated deglutarylation of glucose 6 phosphate dehydrogenase, the rate-limiting enzyme in the PPP, increases its activity and contributes to antioxidant defenses in brain (Zhou et al., 2016). This regulation might also affect purine metabolite levels. In SIRT5^−/−^ cortex, we observed increased abundances of several purine metabolites (adenine, adenosine, xanthine, urea) as well as alterations in some key purine metabolism enzymes, PRPS1 and PPAT, which mediate the initial steps of *de novo* synthesis. Many of the rate-limiting and ATP-dependent reactions within purine metabolism are localized to mitochondria. Studies demonstrate that mitochondrial dysfunction disrupts purine metabolism and can lead to defective DNA repair, RNA synthesis and signaling. Therefore, alterations in purine metabolites in SIRT5^−/−^ cortex might reflect a general state of mitochondrial stress. These stress signals might be responsible for genetic compensation of the affected pathways.

Purine metabolism was the only pathway found common to SIRT5, PKCε and IPC and thus suggests it as a common target of preconditioning paradigms for ischemic tolerance. The brain relies significantly on the salvage pathway of purine synthesis to maintain adenine nucleotides for AMP, ADP and ultimately ATP (Gerlach et al., 1971; Allsop and Watts, 1980). During ischemia, severe metabolic stress induces rapid hydrolysis of ATP (Whittingham et al., 1984), accumulation of AMP, and degradation of AMP into non-salvageable purine nucleosides (Marques et al., 1998; Sala-Newby et al., 2000). Release of purine nucleosides or their further degradation to xanthine and uric acid significantly reduces the post-ischemic total adenine nucleotide and ATP pools. Here, we found that purine metabolites (adenine, adenosine, xanthine, urea) were more abundant in SIRT5^−/−^ cortex, which might indicate impaired salvage and recycling of purines. Increased adenosine in SIRT5^−/−^ could be a sign of stress on the purine system and thus salvage pathway enzymes may be protein targets of SIRT5. Adenosine release is a particularly robust occurrence in ischemic injury (Lynch et al., 1998) and exerts a neuroprotective effect by suppressing glutamatergic transmission through the adenosine A_1_ receptor (A_1_AR) (Rudolphi et al., 1992; Perez-Pinzon et al., 1996). Interestingly, activation of A_1_AR emulates IPC-induced protection from ischemia *ex vivo* whereas inhibition of PKCε reduces its protective effects (Lange-Asschenfeldt et al., 2004). PKCε activation also altered purine metabolites in WT but not SIRT5^−/−^, suggesting SIRT5 as a possible mediator of the PKCε-A_1_AR signaling pathway. Furthermore, of all metabolic genes induced by IPC (Stenzel-Poore et al., 2003), only purine metabolism genes (*POLR2A, AK1, POLA1*) overlapped with metabolites from SIRT5 and PKCε analysis. Together these results point to purine metabolism as a common target of preconditioning-induced protection. The enzymatic targets of SIRT5 in purine metabolism will be a focus of future studies.

In Conclusion, we report for the first time, metabolic pathways regulated by SIRT5 in the brain. While some of these pathways overlapped with known targets of SIRT5 in heart and liver, some are novel including the glutamate-glutamine cycle and purine metabolism. Both of these pathways play an essential role in brain function. Given that Sirtuins are crucial members of the vitagene network that enhances redox homeostasis and healthspan (Calabrese et al., 2008), metabolic regulation by SIRT5 is likely to contribute to the brain's adaptive response to stress and neurodegenerative processes. For example, neuronal SIRT1 (Koronowski et al., 2017) and Nrf2 function in astrocytes (Narayanan et al., 2015) are required for resveratrol-induced protection from stroke in mice. These studies highlight the importance of robust cellular responses to energetic and oxidative stress in combating ischemic injury. Not unlike SIRT1 and Nrf2, SIRT5 is essential for PKCε-induced neuroprotection (Morris-Blanco et al., 2016) and may indeed contribute through enhancement of the aforementioned pathways described here. However, the functional significance of SIRT5-mediated metabolic regulation in brain still needs to be tested, and will certainly be a focus of future investigation. Together, our findings shed light on how sirtuins influence brain metabolism and how their pharmacological modulation can be taken advantage of in the treatment of neurological disease.

## Author contributions

KK, NK, KM-B, HS-C, TG, and MP-P: contributed to the conception, design of the study as well as the acquisition, analysis, and interpretation of data. All authors critically revised this work for intellectual content, approved the final version for publication and are in agreement to be held accountable for all aspects of the work.

### Conflict of interest statement

The authors declare that the research was conducted in the absence of any commercial or financial relationships that could be construed as a potential conflict of interest.

## Abbreviations

SIRT5: silent information regulator two-homolog 5
IPC: ischemic preconditioning
PKCε: protein kinase c epsilon
ΨεRACK: PKCε-specific activator
PCA: principal component analysis
BBB: blood brain barrier
PPP: pentose phosphate pathway
A_1_AR: adenosine A_1_ receptor.
